# Cellular retinoic acid binding protein-II expression and its potential role in skin aging

**DOI:** 10.18632/aging.101813

**Published:** 2019-03-18

**Authors:** Alessandra Bielli, Maria Giovanna Scioli, Federico D’Amico, Chiara Tarquini, Sara Agostinelli, Gaetana Costanza, Elena Doldo, Elena Campione, Daniela Passeri, Filadelfo Coniglione, Augusto Orlandi

**Affiliations:** 1 Anatomic Pathology, Department of Biomedicine and Prevention, Tor Vergata University, Rome, Italy; 2 Dermatology, Department of Internal Medicine, Tor Vergata University, Rome, Italy; 3 Department of Clinical Science and Translational Medicine, Tor Vergata University, Rome, Italy; 4 Department of Biomedical Sciences, Catholic University Our Lady of Good Counsel, Tirana, Albania; * Equal contribution

**Keywords:** skin aging, CRABP-II, retinoids, keratinocyte proliferation and differentiation, dermal remodeling

## Abstract

Skin aging is an intricate biological process consisting of intrinsic and extrinsic alterations of epidermal and dermal structures. Retinoids play an important role in epidermal cell growth and differentiation and are beneficial to counteract skin aging. Cellular retinoic acid binding protein-II (CRABP-II) selectively binds *all trans*-retinoic acid, the most active retinoid metabolite, contributing to regulate intracytoplasmic retinoid trafficking and keratinocyte differentiation. Immunohistochemistry revealed a reduced epidermal and dermal CRABP-II expression in aged human and mouse skin. To better clarify the role of CRABP-II, we investigated age-related skin changes in CRABP-II knock-out mice. We documented an early reduction of keratinocyte layers, proliferation and differentiation rate, dermal and hypodermal thickness, pilosebaceous units and dermal vascularity in CRABP-II knock-out compared with wild-type mice. Ultrastructural investigation documented reduced number and secretion of epidermal lamellar bodies in CRABP-II knock-out compared with wild-type mice. Cultured CRABP-II knock-out-derived dermal fibroblasts proliferated less and showed reduced levels of TGF-β signal-related genes, Col1A1, Col1A2, and increased MMP2 transcripts compared with those from wild-type. Our data strongly support the hypothesis that a reduction of CRABP-II expression accelerates and promotes skin aging, and suggest CRABP-II as a novel target to improve the efficacy of retinoid-mediated anti-aging therapies.

## Introduction

Skin aging is a biological process consisting in two types: intrinsic or chronological aging, and extrinsic aging or photoaging [1]. The latter is mainly due to the progressive and cumulative exposure to ultraviolet radiation [2]. Although pathogenetically distinct, several studies revealed that chronological aging and photoaging share some crucial molecular pathways, particularly in subjects aged over 70 years [3]. Intrinsic aged and photoaged skin are characterized from epidermal atrophy and dermal thinning for the progressive decrease of cellularity, collagen synthesis and the increased collagen degradation from upregulation of dermal activity of matrix metalloproteinases (MMPs) and the reduction of TGF-β signaling [4,5]. Dermal fibroblasts greatly contribute to the production, organization and homeostasis of extracellular matrix (ECM) [6]. Age-related reduced proliferation of fibroblasts further contributes to impair dermal homeostasis and favors age-related skin alterations [7,8]. The critical role of fibroblasts in skin integrity is indirectly confirmed by the promotion of skin regeneration and rejuvenation obtained by experimental autologous injection of cultured dermal fibroblasts [9]. The impairment of TGF-β signaling-related genes is a major contributor for the reduction of ECM protein synthesis in aged skin [10,11]. Human skin converts vitamin A (retinol) to its biologically active metabolite *all-trans* retinoic acid (*at*RA) [12]. The latter contributes to control cell growth and differentiation as well as to maintain skin homeostasis [13]. In particular, *at*RA is known to have a strong effect on growth and differentiation of human epidermal cells *in vivo* and *in vitro* [14-16]. Topical *at*RA administration, used for the therapy of precancerous actinic keratosis, is also applied to counteract skin aging, and likely favors dermal collagen accumulation [17,18]. Efficacy of retinoid therapy was documented in both intrinsic aging and photoaging of skin [19]. Sun-exposed and sun-protected adult skin received equal beneficial effect from *at*RA therapy, with beneficial effects particularly on keratinocyte proliferation and dermal collagen production, whereas neonatal skin was unresponsive [3]. These findings suggest the selective efficacy of retinoid treatment in aged skin [16,19]. Two classes of receptors mediate the biological effects of *at*RA. Classically, retinoids exert their pleiotropic and transcriptional effects through the binding to nuclear receptors, namely the family of retinoic acid receptors (RARα, β, and γ) and retinoid X receptors (RXRα, β, and γ), with specific functions [20,21]. Recent studies documented that also cytoplasmic cellular retinol banding proteins (CRBP-I and CRBP-II) and cellular retinoic acid binding proteins (CRABP-I and CRABP-II) play a crucial role in skin homeostasis and carcinogenesis, likely by contributing to regulate intracytoplasmatic retinoid trafficking [22,23]. CRABP-I is present in many tissues and cells, whereas CRABP-II is recognized as the predominant form of human epidermis [15,24]. In particular, CRABP-II is expressed by fibroblasts and suprabasal keratinocytes, whereas CRABP-I mostly from epidermal melanocytes [24,25]. CRABP-II selectively binds *at*RA and its expression increased after *at*RA treatment, suggesting a sort of intracellular regulatory feedback [26]. Although CRABP-II involvement in skin carcinogenesis is well documented [27], no evidence of its potential role in aging process exists. To this aim, we investigated CRABP-II expression in the skin of aged donors and the effects of CRABP-II loss on skin aging in mice.

## RESULTS

### CRABP-II expression is reduced in human epidermis with aging

**Figure 1:**
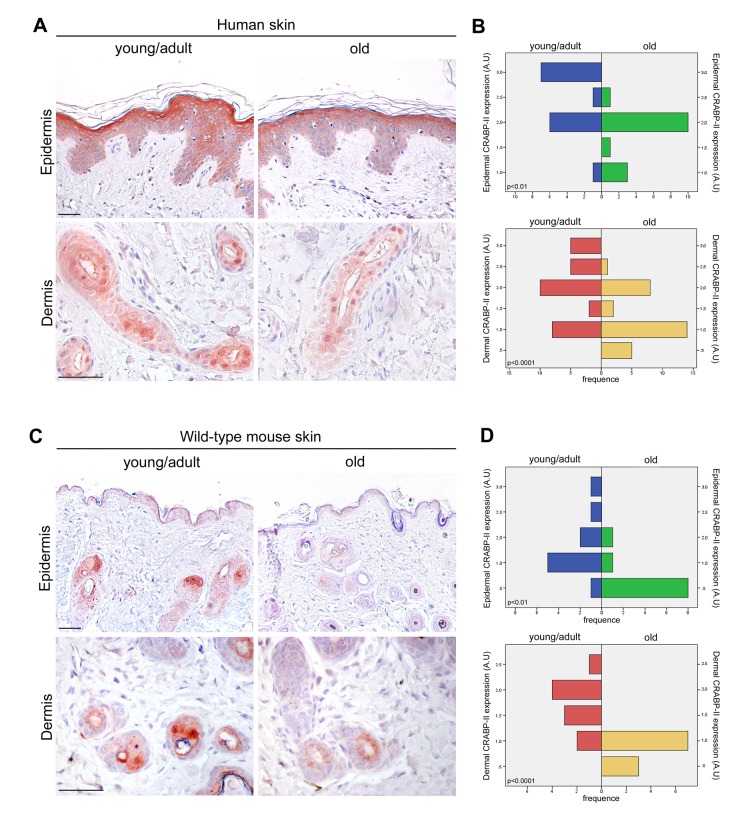
**CRABP-II expression is reduced in aged human and mouse skin.** (**A**) Representative images of CRABP-II immunostaining of human normal skin from young/adult and old donors. (**B**) Bar graphs show semiquantitative evaluation of human epidermal and dermal CRABP-II immunostaining (n=15 young/adult and n=15 old), respectively. (**C**) Representative images of CRABP-II immunostaining of young/adult and old wild-type mouse skin. (**D**) Bar graphs show semiquantitative evaluation of mouse epidermal and dermal CRABP-II immunostaining (n=10 young/adult and n=10 old WT). Scale bar: 50µm. Mann-Whitney’s U-test. Abbreviation: AU, arbitrary units.

To investigate the potential role of CRABP-II in skin aging, immunostaining was performed on paraffin sections of human skin samples from donors of different age. As shown in Fig. 1A, immunohistochemistry documented in young/adult human epidermis a faint cytoplasmic CRABP-II expression in basal and malpighian keratinocytes, a more intense cytoplasmic immunoreaction, also nuclear, in the granular layer, and its absence in the stratum corneum, according to the expression pattern described by others [28–30]. CRABP-II was also strongly expressed from dermal fibroblasts and cutaneous adnexal structures. Semiquantitative evaluation (Figure 1B) of immunohistochemistry documented that CRABP-II expression in old human epidermis and dermis was reduced compared with that of young donors (p<0.01 and p<0.0001, respectively).

### CRABP-II gene deletion accelerates skin aging

**Figure 2:**
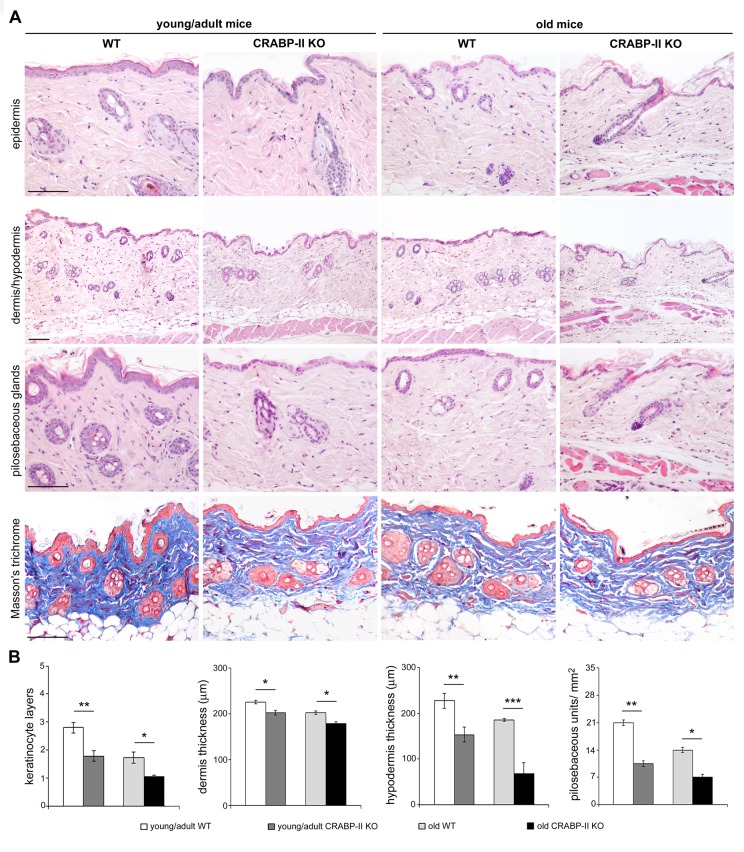
**Skin aging histological features compare earlier and more pronounced in CRABP-II** **knock-out mice.** (**A**) Representative Haematoxylin&Eosin and Masson's trichrome-stained skin sections of young/adult and old wild-type (WT) and CRABP-II knock-out (KO) mice. (**B**) Bar graphs show semiquantitative evaluation of skin aging parameters (n=10 young/adult and n=10 old WT, n=10 young/adult and n=10 old CRABP-II KO). Scale bar: 100µm. Values are group mean ± SEM. *t*-Test: *, ** and *** indicate *p*<0.05, *p* <0.01 and *p*< 0.001, respectively.

**Figure 3:**
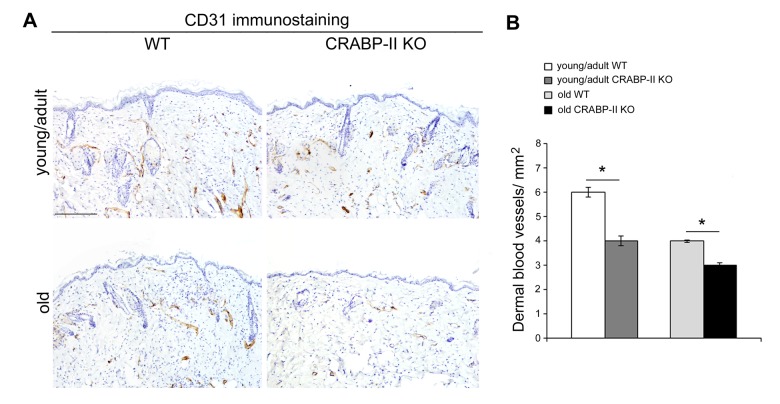
**Dermal blood vessels are reduced in CRABP-II** **knock-out mice.** (**A**) Representative images and (**B**) bar graph showing semiquantitative evaluation of CD31-positive dermal small vessels in young/adult and old wild-type (WT) and CRABP-II knock-out (KO) mice (n=10 young/adult and n=10 old WT, n=10 young/adult and n=10 old CRABP-II KO). Scale bar: 100µm. Values are group mean ± SEM. *t*-Test: * indicates *p*<0.05.

The above data highlight an age-related decrease of CRABP-II expression in human skin with aging. To verify if that decrease was not species-related, we investigated CRABP-II also in mouse skin. Similarly to humans, epidermal and dermal CRABP-II expression was reduced in old compared with young/adult wild-type mice (Figure 1C and D; p<0.01 and p<0.0001). Microscopic and morphometric examination of H&E-stained skin sections (Figure 2A and B) also revealed a decreased number of epidermal layers and a reduction of dermal thickness in young/adult CRABP-II knock-out compared with wild-type mice (p<0.05), that became more pronounced comparing old groups (p<0.01). A hypodermic thinning (p<0.01) and a decrease of pilosebaceous units (p<0.05) were also observed in CRABP-II knock-out compared with wild-type young/adult mice. The reduction of hypodermis thickness and pilosebaceous units was even more marked in old CRABP-II knock-out compared with wild-type mice (p<0.001 and p<0.01, respectively). Masson’s trichrome staining showed thickened collagen bundles in wild-type young/adult mice, instead in wild-type old and CRABP-II knock-out mice collagen bundles decreased and became loose (Figure 2A). CD31 immunostaining (Figure 3A and 3B) showed also a reduction in dermal blood vessels in CRABP-II knock-out mice compared with both young/adult and old with wild-type groups (p<0.05).

### Epidermal proliferation and differentiation are reduced in old CRABP-II knock-out mice

**Figure 4:**
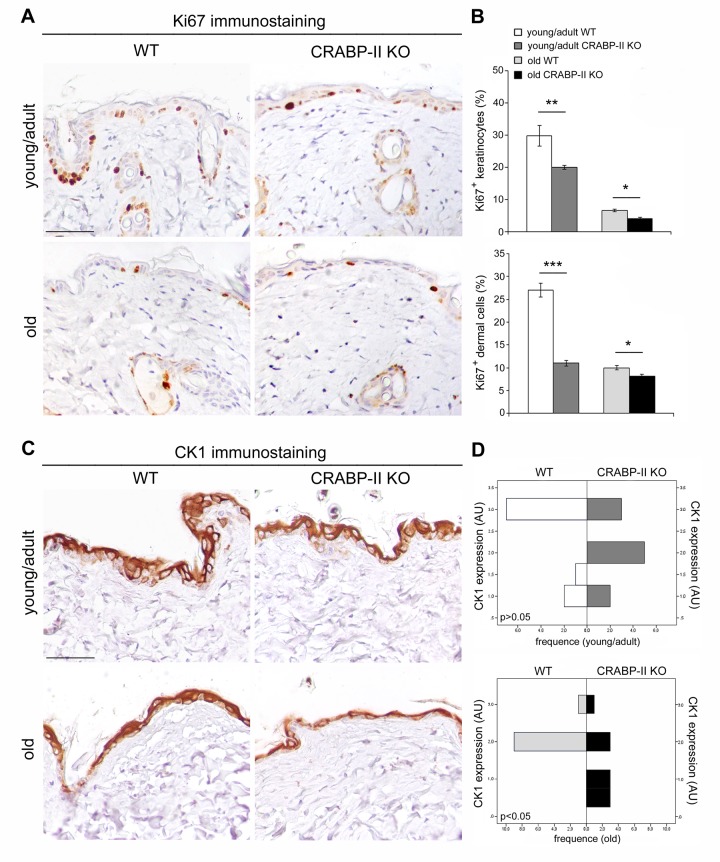
**Epidermal proliferation and differentiation are reduced in CRABP-II** **knock-out mice.** (**A**) Representative images of Ki67 immunostaining of young/adult and old wild-type (WT) and CRABP-II knock-out (KO) skin. Scale bar: 50µm. (**B**) Bar graphs show semiquantitative evaluation of Ki67 positive epidermal keratinocytes and dermal cells (n=10 young/adult and n=10 old WT, n=10 young/adult and n=10 old CRABP-II KO). Values are mean ± SEM. *t*-Test: *, ** and *** indicate *p*< 0.05, *p*< 0.01 and *p*< 0.001, respectively. (**C**) Representative images of cytokeratin 1 (CK1) immunostaining of young/adult and old WT and CRABP-II KO skin. Scale bar: 50µm. (**D**) Bar graphs showing the semiquantitative CK1 evaluation (n=10 young/adult and n=10 old WT, n=10 young/adult and n=10 old CRABP-II KO). Mann-Whitney’s U-test. Abbreviation: AU, arbitrary units.

In order to define if an impaired proliferation contributed to the severity of age-related alterations observed in CRABP-II knock-out mice, we evaluated keratinocyte and dermal cell proliferation *in vivo* by Ki67^+^ immunohistochemistry. Both young/adult and old CRABP-II knock-out showed a reduction of epidermal Ki67^+^ basal keratinocytes compared to age-matched wild-type mice (Figure 4A and 4B; p<0.01 and p<0.05, respectively). Also the percentage of Ki67^+^ dermal cells of young/adult and old CRABP-II knock-out was reduced compared to age-matched wild-type mice (p<0.001 and p<0.05, respectively).

To verify if an impaired differentiation process associated to the reduced keratinocyte proliferation in CRABP-II knock-out mice, we analyzed cytokeratin 1 (CK1) expression, a marker of epidermal differentiation, by immunohistochemistry. As reported in Figure 4C and 4D, epidermal CK1 expression was significantly reduced in old but not in young/adult CRABP-II knock-out compared to wild-type mice counterpart (p<0.05). Altogether, those data indicated that reduced cell proliferation is an early pathophysiological sign of aging, whereas impaired keratinocyte differentiation is a later event in CRABP-II knock-out mice skin.

### Age-related damage of epidermal barrier is increased in CRABP-II knock-out mice

**Figure 5:**
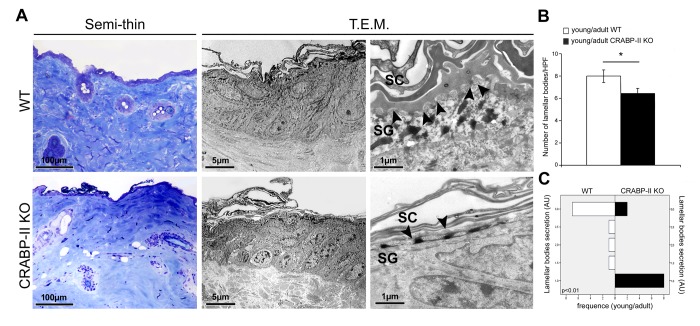
**Ultrastructural evidence of age-related epidermal damage is early and greater in CRABP-II knock-out mice.** (**A**) Representative images of toluidine blue-stained semithin EPON-embedded sections and transmission electron microscopy (T.E.M.) photographs of epidermis of young/adult wild-type (WT) and CRABP-II knock-out (KO) mice. (**B**) Bar graphs show the semiquantitative evaluation of ultrastructural epidermal number of lamellar bodies (n=10 young/adult WT and n=10 young/adult CRABP-II KO). Values are group mean ± SEM, *t*-Test: * indicates *p*< 0.05. (**C**) Semiquantitative evaluation of ultrastructural epidermal secretion of lamellar bodies (n=10 young/adult WT and n=10 young/adult CRABP-II KO). Mann-Whitney’s U-test. Arrow heads indicate lamellar bodies. Abbreviations: SC, stratum corneum; SG, stratum granulosum; HPF, High Power Field; AU, arbitrary units.

One of the most characteristic effects of skin aging is the epidermal reduction of lamellar bodies and their secretion in the extracellular space. To investigate those aspects, we performed ultrastructural analysis of skin. Transmission electron microscopy (T.E.M.) documented a greater reduction of the number and secretion of epidermal lamellar bodies at the stratum granulosum-stratum corneum interface in young/adult CRABP-II knock-out yet compared to wild-type young/adult (Figure 5A and B; p<0.05 and p<0.01, respectively). The difference was maintained in skin samples from old mice (not shown). Those findings documented that age-related damage of epidermal barrier appears earlier and results more severe in CRABP-II knock-out mice.

### CRABP-II loss influences proliferation and transcriptional profile of cultured dermal fibroblasts

**Figure 6:**
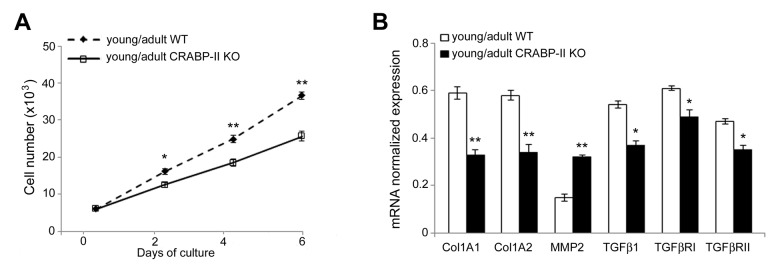
**Proliferation rate and transcriptional profiling are altered in cultured dermal fibroblasts from CRABP-II** **knock-out mice.** (**A**) Growth curve of cultured dermal fibroblasts from young/adult wild-type (WT, n=3) and CRABP-II knock-out mice (KO, n=3). (**B**) Col1A1, Col1A2, MMP2, TGFβ1, TGFβ RI and TGFβ RII mRNA normalized expression in dermal fibroblasts from young/adult WT and CRABP-II KO. Values are group mean ± SEM. *t*-Test: * and ** indicate *p*< 0.05 and *p*< 0.01, respectively.

To verify if the age-related reduction of dermal fibroblast proliferation associated to changes of transcriptional profile, we cultured dermal fibroblasts in a two-dimensional system. As shown in Figure 6A, dermal fibroblasts of CRABP-II knock-out proliferated less compared with those from wild-type mice, in particular at 4 and 6 days after seeding (p<0.001), confirming the *in vivo* results. Then, we investigated the effects of CRABP-II loss on the transcriptional profile of dermal fibroblasts (Figure 6B). We documented a reduction of Col1A1 and Col1A2 (p<0.01), TGFβ1, TGFβRI and TGFβRII (p<0.05), and an increase of MMP2 (p<0.01) transcript levels in cultured dermal fibroblasts from young/adult CRABP-II knock-out compared with those from wild-type mice. These data strongly suggest that age-related effects are, at least in part, mediated by an early alteration of TGFβ-related pathway, a reduction of collagen synthesis and an increased MMP-mediated ECM remodeling.

## DISCUSSION

In this study, we investigated for the first time the role of CRABP-II expression in skin aging. We documented that reduced CRABP-II expression characterizes human and mouse aged skin and loss of CRABP-II gene leads to a premature and a more severe skin aging involving both dermal and hypodermal compartments. CRABPs are a family of proteins that specifically bind *at*RA facilitating its transport into the nucleus where atRA binds its receptors (RARs). CRABP-II, first detected in embryonic and neonatal skin of rats and chicks, is also now recognized as the predominant form in human epidermis [24,25]. *at*RA is known to have a profound effect on growth and differentiation of human epidermal cells both *in vivo* and *in vitro* [15]. CRABP-II has been suggested to be a key marker of retinoid-linked activity in the skin, although its exact function is still partially unknown [16]. Some studies reported that CRABP-II is induced by *at*RA in full-thickness skin, in epidermis and cultured fibroblasts [2,31,32]. In particular, epidermal differentiation, stimulated by culture at the liquid-air interface, tended to enhance CRABP II expression [25]. According to previous studies [28–30], we documented CRABP-II expression in differentiating suprabasal keratinocytes, in particular the granular layer, with a cytoplasmic and nuclear distribution. Since we documented a reduction of CRABP-II expression in aged human and mouse skin, to better clarify its role in skin aging, we investigated the onset and the severity of age-related skin changes in CRABP-II knock-out C57bl/6 mice. We documented an early reduction of epidermal keratinocyte layers, a dermal and hypodermal thinning and a decreased number of pilosebaceous units in young/adult CRABP-II knock-out. This reduction was further enhanced in old CRABP-II knock-out skin when compared with wild-type mice. In addition, Masson’s trichrome staining revealed a better quality of dermal collagen in wild-type young/adult mice that showed collagen bundles more thickened than wild-type old and CRABP-II knock-out mice, whose collagen bundles decreased and became loose. This finding confirmed the decreased collagen content and its degradation observed in aged skin [33].

Since age-related epidermal thinning and dermal atrophy are due to the decrease of keratinocyte and dermal fibroblast turnover rate [16], we analyzed if CRABP-II gene loss influences cutaneous cell proliferation and differentiation *in vivo* by immunohistochemistry. We documented that keratinocytes and dermal fibroblasts in CRABP-II knock-out proliferated less than wild-type starting from young/adult mice, and this reduction got worse comparing old group. Similarly, CK1 expression slightly decreased in young/adult and more markedly in old CRABP-II knock-out mice when compared with wild-type group. Reduced CK1 expression is reported to be a characteristic of skin aging [34,35]. The senescence of transit-amplifying suprabasal cells (CK1 positive) is considered to be responsible for epidermal hypoplasia and epidermal thinning during the late phase of skin aging [36]. Our data support that the age-related changes appear earlier in CRABP-II knock-out old mice with a reduced epidermal and dermal proliferation and successively become more severe, with an impaired keratinocyte differentiation. It has been reported that retinol treatment increased dermal vascularity by stimulating endothelial cell proliferation [37]. The decrease of dermal blood vessels in CRABP-II knock-out compared with wild-type mice strongly suggests that a reduced vascular supply contributes to the age-related epidermal and dermal atrophy [38]. Aged skin shows alterations of permeability and severe dermal sclerosis, resulting an age-related impairment of epidermal barrier [39]. The stratum corneum is the outermost skin layer and provides a highly efficient barrier for water and a protection against infections [40]. Epidermal lamellar bodies are vesicles containing lipids and enzymes present in keratinocytes of stratum spinosum in high number at the interface of the stratum granulosum and stratum corneum [40]. Before extracellular exocytosis, lipids from lamellar bodies are enzymatically metabolized to free ceramides and fatty acids that contribute to the formation of a protective barrier in the stratum corneum [40]. Ultrastructural analysis documented a reduced number of lamellar bodies as well as their secretion in CRABP-II knock-out mice starting from young/adult group, suggesting that the loss of CRABP-II expression favors an early impairment of epidermal barrier function.

Aging promotes aberrant collagen homeostasis by down-regulating type I collagen accumulation, the major structural protein in skin, and promoting collagen degradation [14]. In particular, the alteration of organization, structure and content of dermal collagen derives from the imbalance of its homeostasis, likely due to the elevated activity of matrix metalloproteinases (MMPs) [41]. Imbalance of collagen homeostasis is considered responsible for the winkled appearance and atrophy of aged skin [41]. Vitamin A and its metabolites have been shown to promote new deposition of collagen and prevent its degradation by increasing type I procollagen and reducing MMP-1 activity [10]. In order to clarify if CRABP-II loss also influences dermal collagen homeostasis, we cultured dermal fibroblasts from CRABP-II knock-out and wild-type young/adult mice. Our data confirmed the *in vivo* observation of a reduced proliferation rate in the dermis of CRABP-II knock-out mice. It has been hypothesized that retinoids increase collagen synthesis either by restoring procollagen expression, inhibited by c-Jun signaling triggered by UV irradiation, or by activating TGF-β-mediated signal transduction [8,42], which may in turn induce collagen synthesis in dermal fibroblasts by a paracrine action [42]. In fact, CRABP-II knock-out-derived dermal fibroblasts displayed a different modulation of TGFβ-related pathway, in particular a reduced expression of Col1A1, Col1A2, TGFβ1, TGFβRII and an increase of MMP2 transcripts compared with wild-type mice. Those data strongly suggest that age-related changes in CRABP-II knock-out mice are at least in part mediated by an alteration of TGFβ-related pathway signaling, with a consequent reduction of collagen synthesis and an increased MMP-mediated extracellular matrix degradation.

In conclusion, our data strongly support the involvement of CRABP-II in skin aging, since CRABP-II gene loss accelerated age-related skin changes. However, these findings suggest the need of further human studies on chronological aging and photoaged skin to establish the specific role of CRABP-II and CRABP-II-related retinoid signaling and to develop new targeted therapeutic interventions to counteract skin aging.

## MATERIALS AND METHODS

### Human skin

Anonymous sections of human normal skin were obtained from the paraffin block archive of the Anatomic Pathology, Tor Vergata University of Rome, from patients undergoing skin surgery for non-malignant lesions. Before surgical procedures, written consent was obtained and the study were in accordance with Declaration of Helsinki and local Ethical Committee guidelines. Patients were arbitrary divided in two groups: young/adult, from 18 to 45 years (n=15; mean age 31.1) and old, from 60 to 75 years (n=15; mean age 68.7).

### Generation of CRABP-II null mice and mutant genotyping

Transgenic C57Bl/6 mice heterozygous for CRABPII gene were kindly gifted by Dr. V. Giguère (McGill University/Animal Resources Centre, Montreal, Canada). CRABP-II gene knock-out mice were obtained, as reported [27]. Mice were housed in a temperature-controlled facility with a 12 light/dark cycle, according to the EU guide for the use of laboratory animals and the study approved by Institutional Animal Care and Use Committee, IACUC, according to the Dlgs 116/92. To confirm the mice progenies, DNA was obtained from the terminal portion of the tails by using the manufacture’s extraction protocol (Qiagen, Hilden-Germany, QIAamp-kit-tissue). The genotype was confirmed by reverse transcription PCR analysis, using specific primers [27].

### Microscopic investigation of mice skin

Mice were enrolled in two age groups, 5-10 months (young/adult, n=10) and 18-28 months (old, n=10) for both wild-type and CRABP-II knock-out. Dorsal skin was excised after killing by cervical dislocation and harvested for histological examination. Skin samples were fixed in 10% buffered formalin. Paraffin sections were stained with Haematoxylin&Eosin for morphometric quantification of keratinocytes layers, dermis and hypothermis thickness and pilosebaceous units [43]. The quality and distribution of collagen was evaluated after tissue staining with the Masson’s trichrome technique [33].

### Immunohistochemical study

For immunohistochemistry, tissue samples (n=10 young/adult and n=10 old WT, n=10 young/adult and n=10 old CRABP-II KO) were placed in 10% buffered formalin or zinc fixative for 24 h, dehydrated and embedded in paraffin [27]. Serial 4-μm thick sections were deparaffinized, rehydrated and after antigen retrieval and nonspecific peroxidase blocking, incubated with rabbit polyclonal anti-CRABP-II (A300-809A, 1:300, Bethyl, Texas, USA), rabbit monoclonal anti-Ki-67 (NB500-170, 1:300, Novus Biological, USA), anti-cytokeratin 1 (ab24643, 1:2500, Abcam, UK), rat monoclonal CD31 antibody (5573550, 1:100, BD Pharmingen, San Diego, CA, USA). Positive controls (tissues expressing the antigens) and negative controls (tissues not expressing the antigens and immunoglobulin isotype-matched negative controls) were included. Semiquantitative evaluation of CRABP-II and CK1 staining intensity was performed under a light microscope by two researches, independently, by using a grading system in arbitrary units as follows: absence of positivity (0), focal presence (0.5), weak (1), moderate (2), and strong (3) in at least ten randomly selected fields/mouse [27]. The mean scores from each animal were calculated and analyzed using a non-parametric statistical analysis (see Statistical analysis). Ki67 positivity, as the percentage of positive nuclei/total nuclei/field (at 400X magnification) in basal epidermal layer and dermal fibroblasts, was evaluated in at least ten randomly selected fields/mouse [44]. The number of skin dermal blood vessels (CD31 positive) per mm^2^ was evaluated in at least ten randomly selected fields/mouse at 400X magnification [45,46]. The mean score for every sample was calculated and analyzed (see Statistical analysis). The inter-observer reproducibility was > 95%.

### Ultrastructural study

For transmission electron microscopy, small mouse skin samples (n=10 young/adult and n=10 old WT, n=10 young/adult and n=10 old CRABP-II KO) were fixed in Karnovsky’s solution, processed and embedded in EPON 812 [47]. Thin sections were stained with 0.1% toluidine blue, and ultrathin sections, counterstained with uranyl acetate and lead citrate, and photographed with H-7100FA Hitachi transmission electron microscope (Japan). We took ten images (magnification X 15,000) from each specimen to calculate the number of lamellar bodies using an 8×4 cm sized rectangular grid just below the stratum corneum and counted the lamellar bodies within it; the degree of secretion of lamellar bodies was estimated using a semiquantitative scale (+ = 1, ++ = 2, +++ = 3), as reported [48]. The mean score for every sample was calculated and analyzed (see Statistical analysis).

### Isolation of mouse dermal fibroblasts

For isolation of fibroblasts, wild-type (n=3) and CRABP-II knock-out mice (n=3) were killed by cervical dislocation, soaked in betadine and placed in ice, then washed twice with 70% ethanol. The dermis was stretched in 0.25% trypsin solution for epidermis separation, then dermal samples were cut into small pieces were placed in Dulbecco's modified Eagle’s medium (DMEM) plus 2% HEPES and digested with 0.1% of Collagenase type 1 (Sigma-Aldrich, St. Louis, MO, USA). Dermal fibroblasts were used for experiments between passage 1 and 3.

### Cell growth assay

Dermal fibroblasts were seeded at density of 3x10^3^ cells/cm^2^ into 24-well plates and allowed to attach for 24 hours, starved in DMEM containing 0.1% FBS for 24 hours prior to be cultured in complete growth medium. Then, cells were trypsinized and counted, as reported [49].

### Reverse transcriptase and Real-Time polymerase chain reaction

Total RNA was isolated using the TRIZOL reagent, reverse transcribed and Real-Time polymerase chain reaction performed using iQ^TM^ SYBR® Green Supermix (Bio-Rad, CA, USA) in an iQ5 Multicolour Real-time PCR Detection System (Bio-Rad) [50] using primer sequences for collagen 1 alpha 1 (Col1A1), collagen 1 alpha 2 (Col1A2), transforming growth factor 1 (TGFβ1), TGFRI, TGFRII, matrix metalloproteinase-2 (MMP-2) and CRABP-II listed in Supplementary Table 1. Experiments were performed in triplicate and expression values were normalized to GAPDH levels using the following formula: 2^− (ΔCT)^ [51].

### Statistical analysis

For all analyses, p value < 0.05 was considered significant. For categorical variables (semiquantitative evaluation of CRABP2 and CK1 immunohistochemistry as well as lamellar bodies’ secretion), the Mann-Whitney’s U-test was performed. For the remaining quantitative variables, differences were analyzed by one-way analysis of variance (ANOVA) followed from a Bonferroni post hoc test and using the Student *t*-test. Results were presented as the mean ± SEM (standard error of mean). All quantitative data sets presented here passed the normality tests. Homogeneous variance of the input data was confirmed using an F-test. Statistical analyses were performed with SPSS software (IBM, SPSS Statistics, US. version 23).

## Supplementary Material

Supplementary Table
