# Supplementary material for: Cellular retinoic acid binding protein-II expression and its potential role in skin aging
Source: Aging (Albany NY). 2019 Mar 18;11(6):1619–32. doi: 10.18632/aging.101813 (PMC6461173; doi:10.18632/aging.101813)
Supplement: Supplementary Table [file aging-11-101813-s001.pdf]

SUPPLEMENTARY TABLE

Supplementary Table 1. Primers used for Real-Time PCR.

Gene	Primer sequence	Accession number	Tann (°C)
Col1A1	sense 5′-tgttccctactcagccgtctgt-3′<br>antisense 5′-gagccctcgcttccgtactc-3′	NM_007742.3	60
Col1A2	sense 5′-tggcccatctggtaaagaag-3′<br>antisense 5′-acctttgccaccttgaacac-3′	NM_007743.2	60
TGFβ1	sense 5′-ggagcccgaagcggacta-3′<br>antisense 5′-cgaatgtctgacgtattgaagaaca-3′	NM_011577.1	60
TGFβ RI	sense 5′-tagctgaaattgacctaattcctcg-3′<br>antisense 5′-tgcggttatggcagatatagacc-3′	NM_009370.2	60
TGFβ RII	sense 5′-gggattgccatagctgtcat-3′<br>antisense 5′-tgatggcacaattgtcactg-3′	NM_009371.3	60
MMP-2	sense 5′-agatcttcttcttcaaggaccggtt-3′<br>antisense 5′-ggctggtcagtggcttggggta-3′	NM_008610.2	60
CRABP-II	sense 5′-tgatgaggaagatcgctgtg-3′<br>antisense 5′-ttccactctcccatttcacc -3′	NM_007759.2	60
GAPDH	sense 5′-aactttggcattgtggaagg-3′<br>antisense 5′-cacattgggggtaggaacac-3′	NM_001289726.1	60
